# *Notes from the Field: *Hantavirus Pulmonary Syndrome in a Migrant Farm Worker — Colorado, 2016

**DOI:** 10.15585/mmwr.mm6602a6

**Published:** 2017-01-20

**Authors:** Grace Marx, Kaylan Stinson, Monte Deatrich, Bernadette Albanese

**Affiliations:** ^1^Epidemic Intelligence Service, Division of Scientific Education and Professional Development, CDC; ^2^Tri-County Health Department, Greenwood Village, Colorado.

On August 8, 2016, Tri-County Health Department (Adams, Arapahoe, and Douglas counties) in Colorado was notified of a confirmed case of hantavirus pulmonary syndrome (HPS). The patient was a previously healthy male migrant farm worker aged 25 years, living in farm quarters, and working in vegetable fields for 8 weeks before symptom onset. On July 20, he experienced sudden onset of fever, severe headache, myalgias, cough, and nosebleed. He was evaluated at an emergency department on July 23, where his temperature was 103.9°F (40.0°C), and his physical exam was notable for bronchial breath sounds and pulmonary crackles. Chest radiograph revealed bilateral interstitial infiltrates and small pleural effusions, and thrombocytopenia (47,000/*μ*L) was a noted laboratory finding. The patient was hospitalized for 3 days, required minimal oxygen and supportive care, and survived. Serology obtained on hospital admission was positive for hantavirus immunoglobulin M (IgM) and immunoglobulin G antibodies, with a positive result for Sin Nombre virus (SNV) (the primary etiologic agent of HPS in the United States) IgM at 1:6400, consistent with acute infection (Table).

**TABLE:** Laboratory findings associated with hantavirus pulmonary syndrome and Sin Nombre virus infection in a patient, by specimen collection date — Colorado, July 2016

Clinical specimen and laboratory test	Reference range	Collection date		
		July 23	July 24	July 26
Hantavirus IgM antibodies (ELISA)*	<2.00	—^†^	7.13	—^†^
Hantavirus IgG antibodies (ELISA)*	<2.00	—^†^	10.05	—^†^
Sin Nombre virus IgM antibodies (ELISA)^§^	<1:100	—^†^	1:6400	—^†^
Sin Nombre virus IgG antibodies (ELISA)^§^	<1:100	—^†^	1:100	—^†^
Sin Nombre virus IgM antibodies (ELISA)*^,¶^	<0.80	—^†^	4.83; 4.01	—^†^
White blood cells (10^3^/*μ*L)	4.8–10.8	7	8.9	9.4
Hematocrit (%)	42.0–52.0	49	44	43
Platelets (10^3^/*μ*L)	130–400	47	59	144

**Abbreviations:** ELISA = enzyme-linked immunosorbent assay; IgG = immunoglobulin G; IgM = immunoglobulin M.

* Commercial reference laboratory.

^†^ Data not collected.

^§^ Colorado State Department of Public Health and Environment laboratory.

^¶^ Confirmatory reflex testing was performed twice.

Hantaviruses are a genus within the Bunyaviridae family that can cause HPS, a rare and sometimes fatal respiratory disease in humans. The majority of HPS cases in the United States are caused by SNV, which is primarily transmitted by the deer mouse (*Peromyscus maniculatus*) (*1*). The average incubation period is 1–5 weeks after exposure to infected deer mouse urine or droppings (*2*). HPS typically manifests with fever, myalgias, progressive respiratory insufficiency, thrombocytopenia, and leukocytosis. Treatment is supportive. Approximately 60% of hospitalized patients experience pulmonary edema and respiratory failure and require mechanical ventilation (*3*). HPS case-fatality ratio is 38% (*4*).

Tri-County Health Department performed an environmental assessment of the farm on August 17. The residential dwelling was shared with 12 other male farm workers in a 1,000 square-foot wood frame house. Open food containers were found throughout the house; rodent droppings were observed in the kitchen, cement foundation, and ceiling. The patient reported that during the incubation period, he took daytime naps under trees and in abandoned farm buildings on the property, information that was corroborated by the farm owner and foreman. Those napping areas had evidence of rodent habitation including nesting, burrowing, and rodent runs. None of the other housemates reported an acute respiratory illness during the same exposure period and were not medically evaluated. Tri-County Health Department recommended implementing an integrated pest management program in the residential dwelling and workplace, which the farm owner agreed to execute.

Review of HPS cases in Tri-County Health Department’s jurisdiction during the preceding 2 years revealed a fatal case in a farm worker in November 2014 (Colorado Electronic Disease Reporting System, unpublished data, 2016) on a ranch approximately 50 miles east of the farm described in this report. Environmental assessment of that patient’s farm home in 2014 revealed multiple rodent nests and excrement throughout the rural residential dwellings.

This report highlights the importance of considering HPS in farm workers and in other occupations with risk for rodent exposure either at the workplace or in housing provided by the employer (*5*,*6*). Nationally, 23% of reported HPS cases with a reported occupation were working in agriculture (Dr. Annabelle de St. Maurice, CDC, personal communication, 2016). The lack of a vaccine or specific treatment for HPS underscores the importance of focusing on behavioral and environmental risk reduction to prevent SNV infections, including for at-risk occupations, such as farming. Adding supplemental questions to the national HPS case report form* about occupational exposures, including occupation, industry, workplace, and work-related housing or other outdoor activities, will improve identification of work practices and characteristics that increase risk for SNV exposure. Rapid public health assessment of environmental exposure to SNV is critical to mitigate ongoing hazards.

## Footnotes

* https://www.cdc.gov/hantavirus/pdf/hps_case-report-form.pdf.
